# Ethylene-Inducible AP2/ERF Transcription Factor Involved in the Capsaicinoid Biosynthesis in *Capsicum*

**DOI:** 10.3389/fpls.2022.832669

**Published:** 2022-03-03

**Authors:** Jinfen Wen, Junheng Lv, Kai Zhao, Xiang Zhang, Zuosen Li, Hong Zhang, Jinlong Huo, Hongjian Wan, Ziran Wang, Haishan Zhu, Minghua Deng

**Affiliations:** ^1^Faculty of Architecture and City Planning, Kunming University of Science and Technology, Kunming, China; ^2^College of Horticulture and Landscape, Yunnan Agricultural University, Kunming, China; ^3^Institute of Vegetables, Zhejiang Academy of Agricultural Sciences, Hangzhou, China

**Keywords:** *CcERF2*, pepper, capsaicinoids, ethylene, VIGS

## Abstract

Ethylene is very important in the process of plant development and regulates the biosynthesis of many secondary metabolites. In these regulatory mechanisms, transcription factors (TFs) that mediate ethylene signals play a very important role. Capsaicinoids (CAPs) are only synthesized and accumulated in *Capsicum* species, causing their fruit to have a special pungent taste, which can protect against attack from herbivores and pathogens. In this study, we identified the TF *CcERF2*, which is induced by ethylene, and demonstrated its regulatory effect on CAPs biosynthesis. Transcriptome sequencing analysis revealed that the expression patterns of *CcERF2* and multiple genes associated with CAPs biosynthesis were basically the same. The spatiotemporal expression results showed *CcERF2* was preferentially expressed in the placenta of the spicy fruit. Ethylene can induce the expression of *CcERF2* and CAPs biosynthesis genes (CBGs). *CcERF2* gene silencing and 1-methylcyclopropene (1-MCP) and pyrazinamide (PZA) treatments caused a decrease in expression of CBGs and a sharp decrease in content of CAPs. The results indicated that *CcERF2* was indeed involved in the regulation of structural genes of the CAPs biosynthetic pathway.

## Introduction

Peppers (*Capsicum* spp.) are important vegetables worldwide. Pepper fruits are diverse in color, rich in nutrients, have a special pungency and aroma, and so are widely used as food additives (Ou et al., 2018). The pungency of pepper fruit is derived from capsaicinoids (CAPs), which are only biosynthesized in *Capsicum* plants (Iwai et al., 1979; Liu et al., 2019). The CAPs have the effect of curbing attack by herbivores and microorganisms. At the same time, CAPs are also widely used in many fields such as food, medicine, cosmetics, and agricultural pest control; CAPs are also widely used in riot prevention, personal defense, and in the military and national defense (Lejeune et al., 2007; Tewksbury et al., 2008; Ludy et al., 2012). The CAPs are synthesized and accumulated in fruit placental tissues (Iwai et al., 1979). So far, more than 22 CAPs have been identified in peppers. The most important components of CAPs are capsaicin (CaP) and dihydrocapsaicin (DhCaP), and these represent approximately 90% of the total content of CAPs. The accumulation of CAPs is affected by many factors, including endogenous (for example, variety and developmental stage of the fruit) and exogenous factors (for example, light, temperature, water conditions, and biological stress; Phimchan and Techawongstien, 2012; Garruña-Hernández et al., 2013). Genotype is the most important determinant of CAPs content (Bosland et al., 2012; Liu et al., 2017); among the five domesticated species of *Capsicum*, *Capsicum chinense*, and *Capsicum frutescens* accumulate significantly higher contents of CAPs (Deng et al., 2009; Bosland et al., 2012). It is reported that factors such as plant growth regulators, chemicals, temperature, light, and drought stress can change the content of CAPs (Gurung et al., 2011).

The CAPs are synthesized by the fusion of phenylpropane and branched-chain fatty acid pathways in the placenta (Aluru et al., 2003; Blum et al., 2003; Mazourek et al., 2009). The CAPs biosynthesis genes (CBGs) that have been determined to be involved in biosynthesis of CAPs include *PAL*, *Ca4H*, *4CL*, *BCAT*, *Kas*, *FatA*, *ACL*, *ACS*, *CoMT*, *pAMT*, and *CS* (Abraham-Juárez et al., 2008; Liu et al., 2013). The biosynthetic mechanism of CAPs has been widely elucidated using bioinformatics analysis and multi-omics (Qin et al., 2014). As CAPs have considerable application value and commercial use, and much effort has gone into enhancing their content. However, their biosynthesis greatly changes spatiotemporally, and expression of CBGs is precisely regulated at the transcriptional level. Manipulating the expression level of crucial CBGs seems to determine increasing the content of CAPs (Abraham-Juárez et al., 2008; Sun et al., 2019; Zhu et al., 2019). In highly spicy peppers, the transcription level of CBGs (such as *pAMT*, *Kas*, and *CS*) is always higher than that of less spicy varieties (Abraham-Juárez et al., 2008). It is very important that manipulating the expression levels of some transcription factors (TFs) usually change the transcription levels of all genes in the metabolic pathway, thereby affecting the final content of the compound.

For example, the TFs *CaMYB31*, *CaMYB108*, *CaMYB48*, *Erf*, and *Jerf* are associated with regulating the content of CAPs (Keyhaninejad et al., 2014; Phimchan et al., 2014; Arce-Rodríguez and Ochoa-Alejo, 2017; Sun et al., 2019; Zhu et al., 2019). It was found that the Solanaceae-specific TF *MYB31* affects the accumulation of CAPs by directly targeting CBGs, resulting in changing the expression level of CBGs (Zhu et al., 2019). The TF *CaMYB108* activates the CBG promoters, especially those of *COMT*, *pAMT*, and *KasI*, and promotes enhanced expression of CBGs, thereby promoting the biosynthesis of CAPs (Sun et al., 2019). By directly binding and regulating the expression of CBGs, TF *CaMYB48* participates in the biosynthesis of CAPs, but the transcriptional regulation of CAPs biosynthesis has not been fully clarified (Sun et al., 2020). Therefore, it is necessary to identify TFs associated with the biosynthesis of CAPs.

Ethylene plays a significant role in the plant life process (Hu et al., 2020). After synthesis of ethylene, it binds to the receptor ETR and transmits the signal to the nucleus through MAPKK and EIN2 (Gray, 2004). The EIN2 binds to EIN3/EIL1 and EIN3/EIL1 binds to the *ERF1* promoter. The ERF1 binds to the GCC-box-containing genes in the downstream promoter region specifically and their expression can promote secondary metabolism product synthesis (Fujimoto et al., 2000; Brown et al., 2003; Paul et al., 2020). For example, in tobacco, ERF189 and ERF163 can bind to the GCC-box in the promoter region of the tobacco nicotine synthesis-related gene *PMT2* specifically, and directly promote synthesis of tobacco alkaloids (Shoji et al., 2010). The B3 subfamily proteins ORCA3 and ORA59 of the ERF family are two typical TFs associated with the regulation of secondary metabolites. The ORCA3 can upregulate the expression level of indole alkaloid synthesis-related genes in terpenoids, and promote the terpenoid indole biosynthesis (van der Fits and Memelink, 2000, 2001). *Lithospermum erythrophyllum LeERF-1* affects the secondary metabolites of shikonin positively through a mechanism similar to that of ORCA3 in affecting secondary metabolites (Zhang et al., 2011). In *Catharanthus roseus*, *CrERF5* upregulates the biosynthesis and accumulation of bisindole alkaloids (Pan et al., 2019).

The TFs and key enzymes in ethylene synthesis and its signal transduction can promote the biosynthesis of secondary metabolites. In many cases (plants, cells, and hairy roots), the addition of ethylene, addition of 1-aminocyclopropane 1-carboxylic acid (ACC), and overexpression of related TFs can increase the biosynthesis of secondary metabolites (Pan et al., 2000; Gantet and Memelink, 2002; Zhao et al., 2004; Buer et al., 2006; De Boer et al., 2011). In previous studies, *PAL* genes possessed a homolog of the GCC-box in their promoters and *ERF* genes could combine with their cis-acting element (Ohme-Takagi and Shinshi, 1995; Keyhaninejad et al., 2014). Both *Erf* and *Jerf* in pepper have been proposed to be involved in accumulation of pungency (Keyhaninejad et al., 2014). Many of the ERF family are TFs that are candidates for regulating CAP biosynthesis (Song et al., 2020). However, how CAP biosynthesis is regulated at the transcription level is still unknown in peppers.

In this paper, the ethylene-induced AP2/ERF TF *CcERF2*, which was particularly expressed in the placenta was identified. We confirmed that *CcERF2* was associated with CAPs biosynthesis.

## Materials and Methods

### Experimental Materials

The *C. chinense* inbred line SL08 has a high CAPs content and was derived from Shuan La, the hottest pepper genotype in China (Deng et al., 2009). The *Capsicum annuum* inbred line H19 has a low CAPs content, and was derived from Xiangtan Chi Ban Jiao. Pepper seeds germinated in the soil of cell plastic flats in complete darkness at 28°C. The seedlings were grown in a greenhouse of Yunnan Agricultural University on campus under normal conditions.

### Extraction and Detection of CaP and DhCaP

The CaP and DhCaP were extracted and detected according to Deng et al. (2009). The total CAPs content was calculated as (CaP + DhCaP)/0.91 (Deng et al., 2009). Fruits in different development stages of SL08 and H19 were used as material for extraction and detection of CaP and DhCaP. All experiments were repeated three times.

### RNA-Seq and Analysis

RNA-seq was performed according to Liu et al. (2012). Fruits of SL08 at 4, 14, 24, 34, 44, and 54 days after pollination (DAP) were used to isolate total RNA. All of RNA-seq data generated in this study are available from the NCBI Short Read Archive (SRA, BioProject ID: PRJNA789050), and the raw RNA-seq data are freely available at https://www.ncbi.nlm.nih.gov/bioproject/PRJNA789050.

### Exogenous Substance Treatments

Fruit at 24 DAP was used to investigate the effect of different exogenous substances on expression level of *CcERF2* and CBGs after 0.5, 1.0, 1.5, 3.5, 5.5, 8.5, and 11.5 h. Treated with exogenous substances, the 24-DAP fruits of SL08 were soaked in sterile water with 0.01 g/L 6-benzylaminopurine (6-BA), 20 g/L PEG6000, 30% H_2_O_2_, 0.17 g/L gibberellic acid (GA_3_), 0.14 g/L salicylic acid (SA), 17.53 g/L NaCl, 90 g/L glutamate (Glu), or 0.1 mmol/L ethephon (with sterile water as control) under normal conditions (16 h of light at 30 ± 2°C and 8 h of darkness at 20 ± 2°C). After treatment, all fruits were frozen in liquid nitrogen and immediately stored at −80°C for gene expression analysis. All experiments were repeated three times.

### *CcERF2* Cloning and Bioinformatics Analysis

The open reading frame of *CcERF2* was cloned according to Deng et al. (2012). The primers used in the experiment are shown in Supplementary Table S1. The BLAST-protein-nucleic acid (BLASTP) analysis was performed on *CcERF2* through a database^1^; PSORT Prediction was performed for subcellular location prediction; and MEME was used to analyze the amino acid sequence motif. The Portparam tool was used to predict the physical and chemical properties of the protein. SignalP-5.0, TMHMM, and Nepos were used for signal peptide, transmembrane structure, and phosphorylation site of the protein prediction, respectively; SOPMA was used to predict its secondary structure; and ClustalX was used for sequence alignment and phylogenetic analysis. The MEGA 6.0 software was used to construct a phylogenetic tree based on the amino acid sequence through the neighbor joining method, and the bootstrap method was used to evaluate the reliability of each node in the tree, repeated 1,000 times.

### Subcellular Localization and Transcriptional Activation Analysis

Subcellular localization and transcriptional activation analysis were performed according to Zhu et al. (2019) and Sun et al. (2020). The full-length coding sequences of *CcERF2* was cloned into the pAN580 (green fluorescent protein, GFP) vector and fused to the N-terminus of GFP under the control of the CaMV 35S promoter. The constructs were separately introduced into tobacco protoplasts for transient expression, and the GFP fluorescence signals were detected using a Zeiss lsm710 confocal laser scanning microscope (Carl Zeiss Inc., Jena, Germany).

### Gene Expression Analysis

The RNAiso Plus (Takara, Dalian, P. R. China) was used to extract total RNA from pepper fruit. The synthesis of cDNA first strand refers to the instructions of the High Fidelity PrimeScript® RT-PCR Kit (Takara). According to the sequence of related genes revealed by transcriptome sequencing data, specific primers were respectively designed, and the internal reference was the *β-ACTIN* gene. Quantitative real-time PCR (qRT-PCR) was performed according to the method provided by the SYBR®Premix Ex TaqTM II (Tli RNaseH Plus) kit (Takara). The primers used in this study are shown in Supplementary Table S1.

### VIGS Analysis

A fragment of the *CcERF2* coding sequence (CDS) with low similarity to other genes was cloned into pTRV2 and generated the silencing vector pTRV2–*CcERF2*. The VIGS were carried as reported (Zhu et al., 2019). In short, the pTRV2–*CcERF2* and pTVR1 vectors were co-injected into the cotyledon stage seedlings of line SL08. The empty vectors pTRV2 and pTVR1 were co-infiltrated as a control, and pTRV2–PDS and pTVR1 were co-infiltrated as a technical control. The RNA isolated from 24-DAP fruits was used for expression analysis, and 34-DAP fruits were used for CaP and DhCaP measurement. The primer information used in this study is shown in Supplementary Table S1.

### Effects of 1-Methylcyclopropene and Pyrazinamide on Expression of CBGs and Content of CAPs

The SL08 pepper fruit of 24 DAP was used to study the effects of 1-methylcyclopropene (1-MCP) and pyrazinamide (PZA) on expression of CBGs and content of CAPs. The 1-MCP treatment follows: 1 μl L^−1^ 1-MCP fumigated for 12 h and kept in darkness at a temperature of 20 ± 2°C and humidity of 85 ± 5%. The PZA treatment follows: 100 mM PZA was sprayed onto the surface of the fruit until there were droplets, and kept in darkness at a temperature of 20 ± 2°C and humidity of 85 ± 5%. The fruits after 5 days of treatment were stored in liquid nitrogen and used for subsequent analysis. All experiments were repeated three times.

### Ethylene Release Rate Determination

Ethylene release rate was determined according to Xu et al. (2018).

### Statistical Analyses

Each experiment contained three biological replicates and three technical replicates. The error bars indicate SEs. One-way ANOVA was performed to identify significant differences. The relative gene expression was calculated using the 2^−ΔΔCT^ method (Livak and Schmittgen, 2001). Control expression was without induction at the 0 h time point.

## Results and Analysis

### Accumulation of CAPs

Accumulation of CaP and DhCaP was detected in both lines, but the content of CaP was much higher (about 3–4 times) than that of DhCaP. The CaP and DhCaP contents were much higher in the placenta of SL08 than in H19 (Supplementary Figure S1). Both CaP and DhCaP began to accumulate at 9 DAP in the placenta of SL08, reached a peak at 44 DAP, and then began to decline. Both CaP and DhCaP began to accumulate at 11 DAP in the placenta of H19, and began to decrease at 49 DAP. The CaP, DhCaP, and total CAPs contents in the placenta of SL08 were 44.4, 63.1, and 48.0 times those in H19, respectively.

### Expression of *CcERF2* and CBGs Is Consistent With CAPs Biosynthesis

**Figure 1:**
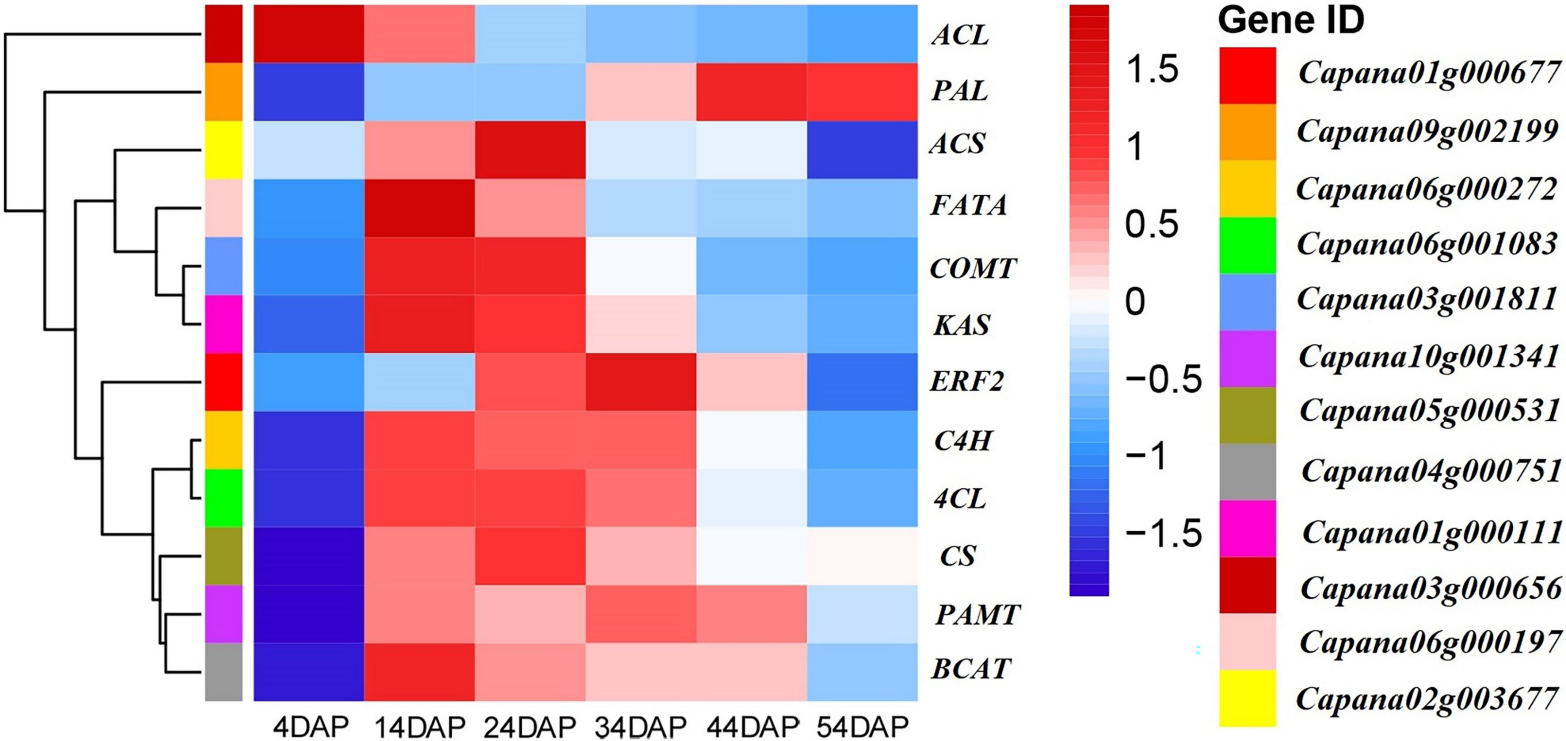
Heat map indicating the capsaicinoids (CAPs) biosynthesis genes (CBGs) and *CcERF2* expression patterns in fruit of inbred line SL08. The heatmap was generated by transcriptome sequencing data with R software packages. The data were normalized in each row, and the numbers on the right side of the figure indicate the gene expression level corresponding to color.

Expression patterns of CBGs during development stages of the placenta were analyzed using transcriptome sequencing data of the placenta and pericarp of line SL08 during the early stage of the laboratory study (Figure 1). Expressions of *CcPAL*, *CcCa4H*, *Cc4CL*, *CcCoMT*, *CcpAMT*, *CcCS*, *CcBCAT*, *CcKAS*, *CcACL*, *CcFAT*, and *CcACS* genes were positively associated with accumulation of CAPs in SL08. The expression of these genes (except *CcACL*) showed a low expression level in fruit at 4 DAP, increased, and then decreased. Gene *CcCS* (*Capana05g000531*) was not expressed in 4-DAP fruit, and reached a maximum in 24-DAP fruit. Expression of *CcCoMT* (*Capana03g001811*), *CATCcBCAT* (*Capana04g000751*), *CcKAS* (*Capana01g000111*), and *CcFATA* (*Capana06g000197*) reached their highest values in 14-DAP fruit. Expression of *CcCa4H* (*Capana06g000272*) and *CcpAMT* (*Capana10g001341*) genes reached their maximum in 34-DAP fruit. Expression of *CcPAL* (*Capana09g002199*) reached its highest value in 44-DAP fruit.

Based on the transcriptome sequencing data of lines SL08 and H19 at different developmental stages of placenta completed in the laboratory, combined with the changes in the CaP and DhCaP contents of the placenta of both lines (Supplementary Figure S1), the AP2/ERF TF *CcERF2* (*Capana01g000677*) was identified. Its expression pattern was similar to that of CBGs (Figure 1). Expression of *CcERF2* was almost undetectable in the early stage of placenta (4 DAP), then rose rapidly, reached a maximum at 34 DAP, and then decreased.

**Figure 2:**
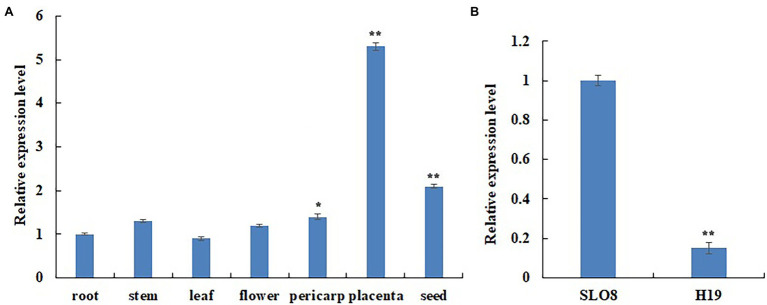
Differential expression assays of *CcERF2*. **(A)** Differential expression assays of *CcERF2* in different tissues of pepper inbred line SL08; Roots, stems, leaves and flowers were collected from 30-day-old seedlings. Fruits at 24 days after pollination (DAP) was collected and was divided into pericarps, placentas and seeds. The relative expression of the root was set to 1, and that of all the other tissues was measured relative to that of the root. **(B)** Differential expression assays of *CcERF2* in pepper inbred line SL08 and H19 fruits. Fruits at 24 DAP was collected to investigate the *CcERF2* expression. The relative expression of the SL08 was set to 1, and that of H19 was measured relative to that of the SL08. The experiments were replicated three biological times and three technical times. Data are expressed as the mean ± SD (*n* = 9). Student’s *t*-test was used to identify significant differences compared to the control (^*^p < 0.05, ^**^p < 0.01).

We measured the expression level of *CcERF2* in some tissues of line SL08 and found that it was mainly expressed in the placenta (Figure 2A). It also showed considerable level of expression in seeds and pericarp, meaning that it has a role in these tissues. We also investigated *CcERF2* expression in the placenta of both lines, and found significantly higher expression in SL08 than in H19 (Figure 2B). Expression of *CcERF2* in the placenta had the same pattern as the contents of CaP and DhCaP. Thus, our results indicated that *CcERF2* may play a vital role in CAPs biosynthesis.

### *CcERF2* Gene Cloning and Bioinformatics Analysis

Based on our transcriptome sequencing data of SL08 and the pepper genome data in the public database, specific primers were designed to clone the full-length CDS of *CcERF2* from SL08. Nucleotide sequence analysis showed that *CcERF2* length was 795 bp. The molecular formula of CcERF2 is C_1328_H_2050_N_376_O_409_S_9_, molecular weight is 30,115.70 D, and theoretical isoelectric point is 5.74. It is a fat-soluble, hydrophilic, and unstable protein. CcERF2 had no signal peptide and no transmembrane structure, and is located in the nucleus (88.8% probability).

**Figure 3:**
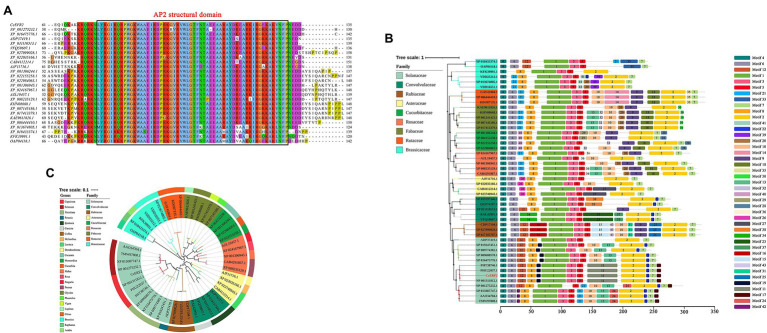
Phylogenic tree of ERF transcription factors (TFs), analysis of promoter cis-acting elements and multiple amino acid sequence alignment of CcERF2 TFs. **(A)** Multiple amino acid sequence alignment of ERF2 TFs. **(B)** Promoter cis-acting elements comprising *CcERF2* in *Capsicum chinense* and 47 *ERF2* promoters in plant. **(C)** Phylogenetic tree comprising CcERF2 in *C. chinense* and 47 ERF2 TFs in plant.

**Figure 4:**
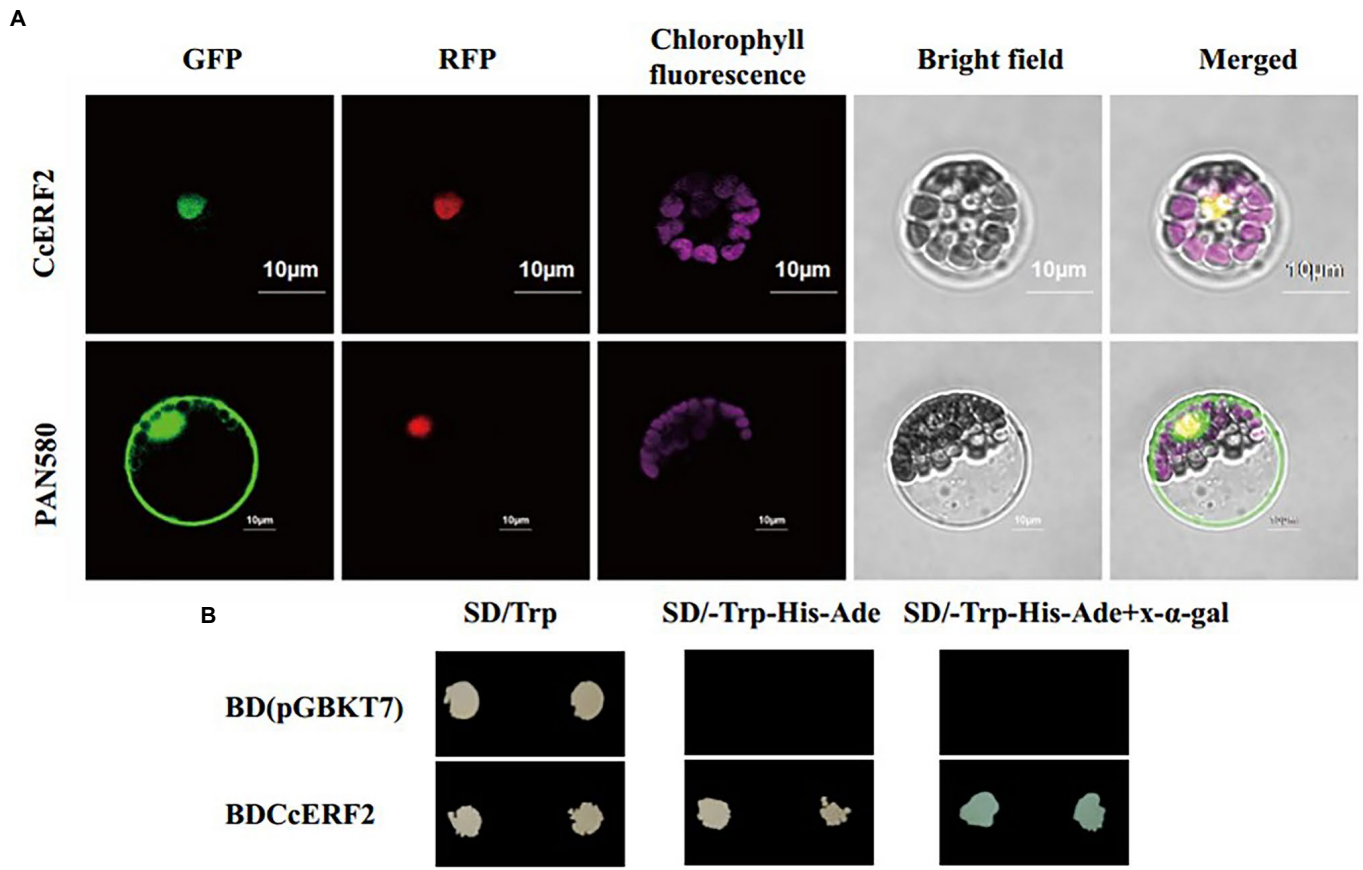
Subcellular localization and transcriptional activation analysis. **(A)** Green fluorescent protein (GFP) signals indicated that CcERF2 was localized to nucleus. GFP fluorescence signals were detected using a Zeiss lsm710 confocal laser scanning microscope (Carl Zeiss, Inc., Jena, Germany). Scale bars: 10 μm. **(B)** Auxotroph plates of SD/–Leu–His–Ade (middle) and SD/–Leu–His–Ade–x-α-gal (right) showing transcriptional activation of protein. SD/Trp, medium lacking tryptophan; SD/-Trp-His-Ade, medium lacking tryptophan, histidine, and adenine.

The protein domain prediction results showed that *CcERF2* belonged to the AP2 superfamily, and the conservative amino acid sequence position was 74–131 (LYRGIRQRPWGKWAAEIRDPRKGVRVWLGTFNTAEEAARAYDKEARKIRGEKAKVNFP; Figure 3A). This domain specifically bound to the 11-bp GCC-box of the ethylene response element and was essential for the ethylene response. Based on BLAST, 48 amino acid sequences including CcERF2 were obtained, and 43 motifs were obtained after motif significance test and analysis. On the whole, the predicted motifs differed within the same family, but the conserved motifs in the same subgroup were almost the same; that is, the closer the related species, the more similar were the motifs. For peppers, they all contained 13 conserved motifs: 1–8, 11, 12, 17, 19, and 22. Of these, motifs 1, 3, and 5 covered the AP2 conserved structure domain (Figure 3B). The secondary structure of CcERF2 protein contained 73 alpha helices (27.56%), 22 extension chains (8.42%), and nine beta turns (3.41%). There were 160 random coils (60.61%). The evolutionary relationship of amino acid sequences showed that the selected ERF sequences were grouped together in different families. This meant that they were conserved in the evolutionary relationship. In genetic relationship, CcERF2 protein was the closest to Solanaceae. In the Solanaceae, it was closest to *Capsicum* and *Solanum*, and most distant to *Petunia* (Figure 3C). The phosphorylation sites of CcERF2 protein were predicted, and 19 amino acid residue types were screened: six types of T, three types of Y, and 10 types of S. The CcERF2 was localized to the cell nucleus (Figure 4A) and the CcERF2 protein had strong activation activity (Figure 4B).

### *CcERF2* Is a TF Induced by Ethylene

**Figure 5:**
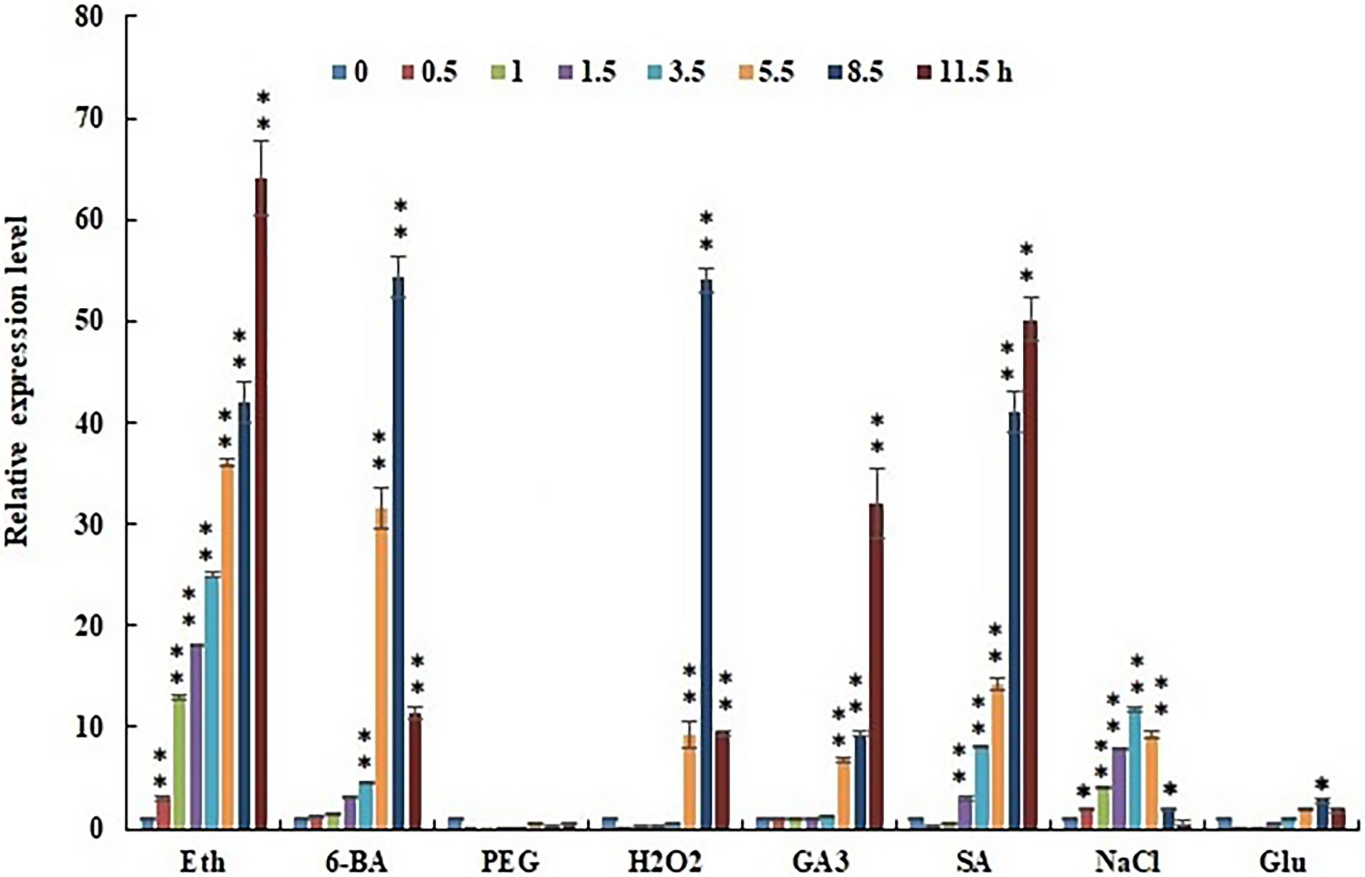
Effect of elicitors (Eth, 6-BA, PEG, H_2_O_2_, GA_3_, SA, NaCl, and Glu) on *CcERF2* expression. The 24 DAP pepper fruits were treated with elicitors (Eth, 6-BA, PEG, H_2_O_2_, GA_3_, SA, NaCl, or Glu), and the fruit were sampled after elicitation for 0.5, 1.0, 1.5, 3.5, 5.5, 8.5, and 11.5 h. Without induction at the 0 h time point was used as control expression. The experiments were replicated three biological times and three technical times. Data are expressed as the mean ± SD (*n* = 9). Student’s *t*-test was used to identify significant differences compared to the control (^*^p < 0.05, ^**^p < 0.01).

Exogenous substances were applied to the 24-DAP fruit of SL08 to test the regulation model. Expression of *CcERF2* in placental tissues was analyzed using qRT-PCR (Figure 5).

The fruits were treated with ethephon, which significantly induced *CcERF2* expression. Compared with the control, *CcERF2* expression increased by 64 times within 11.5 h of applying ethephon. At the same time, the effects of exogenous 6-BA, PEG, H_2_O_2_, GA_3_, SA, NaCl, and Glu on *CcERF2* expression were also tested. Of these, 6-BA, H_2_O_2_, GA_3_, SA, and NaCl significantly induced *CcERF2* expression. Compared with the control, these exogenous substances increased *CcERF2* expression; however, the modes of induced expression were not the same. In the test time range, under the 6-BA, H_2_O_2_, and NaCl treatments, the *CcERF2* expression showed a trend of initial increase and then decrease; however, under GA_3_ and SA treatment, *CcERF2* expression showed an increasing trend. Treatment with Glu had no significant effect on *CcERF2* expression and PEG treatment was inhibitory.

### Effect of Ethephon on Expression of CBGs

**Figure 6:**
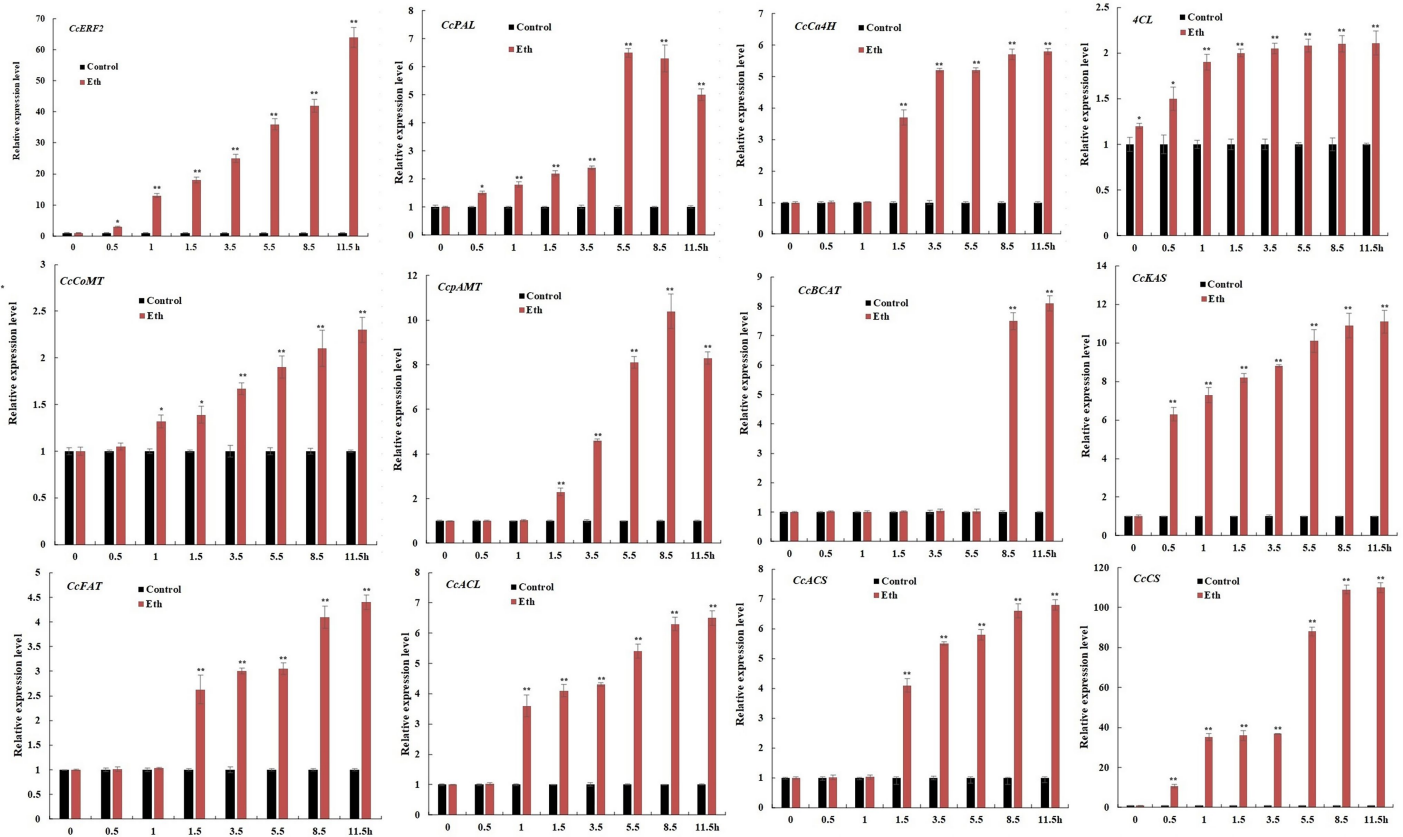
Expression profiles of CBGs after treatment with Eth. The 24 DAP pepper fruits were treated with Eth, and the fruit were sampled after elicitation for 0.5, 1.0, 1.5, 3.5, 5.5, 8.5, and 11.5 h. Without induction at the 0 h time point was used as control expression. The experiments were replicated three biological times and three technical times. Data are expressed as the mean ± SD (*n* = 9). Student’s *t*-test was used to identify significant differences compared to the control (^*^p < 0.05, ^**^p < 0.01).

Ethephon treatment could significantly increase the expression level of CBGs, but the induction effect was inconsistent. Compared to controls, within 11.5 h of applying ethephon, gene expression levels of *CcPAL*, *CcCoMT*, and *CcFAT* were increased about 2.3–5.0 times; of *CcCa4H*, *CcpAmt*, *CcBCAT*, *CcACL*, and *CcACS* were increased about 5.8–8.3 times; of *CcKAS* was increased about 11.1 times; and of *CcCS* was increased more than 100 times. Considering that ethephon significantly induced the expression of *CcERF2*, our results showed that *CcERF2* was a key factor in ethylene-mediated biosynthesis of CAPs (Figure 6).

### Effects of 1-MCP on CBGs Expression and CAPs Contents

**Figure 7:**
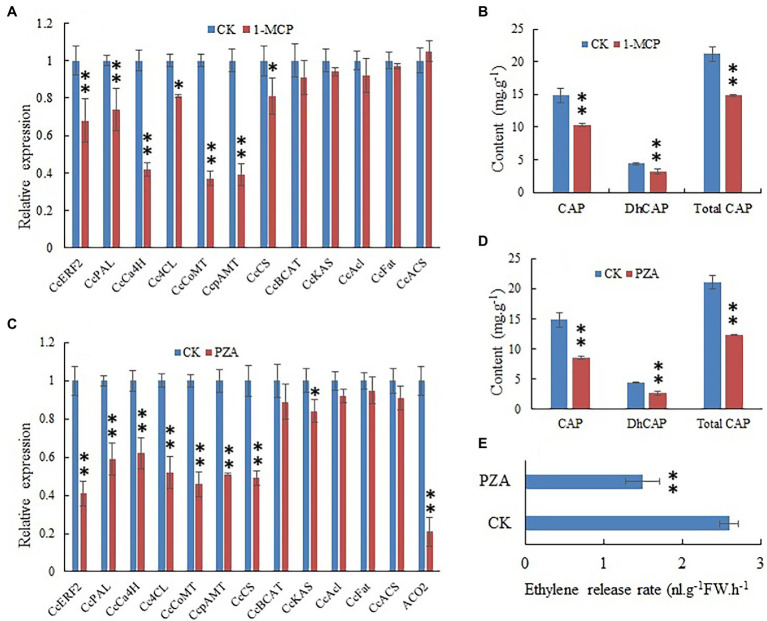
Expression level of CBGs and *CcERF2*, relative contents of capsaicin (CaP), dihydrocapsaicin (DhCaP), and CAPs in 1-methylcyclopropene (1-MCP) and pyrazinamide (PZA) treatment fruit. **(A)** Expression level of CBGs and *CcERF2* in the 1-MCP treatment fruit. Fruits at 24 DAP was sampled to perform the expression level of CBGs and *CcERF2*. The relative expression level of the CBGs and *CcERF2* in the control were set to 1, and that of in 1-MCP treatment fruit were measured relative to that in the control. **(B)** Relative contents of CaP, DhCaP, and CAPs in the 1-MCP treatment fruit at 29 DAP. **(C)** Expression level of CBGs, *CcERF2*, and *ACC oxidase-2* (*ACO2*) in the PZA treatment fruit. Fruits at 24 DAP was sampled to perform the expression level of CBGs and *CcERF2*. The relative expression level of the CBGs and *CcERF2* in the control were set to 1, and that of in PZA treatment fruit were measured relative to that in the control. **(D)** Relative contents of CaP, DhCaP, and CAPs in the PZA treatment fruit at 29 DAP. **(E)** Ethylene release rate in the PZA treatment fruit at 29 DAP. The experiments were replicated three biological times and three technical times. Data are expressed as the mean ± SD (*n* = 9). Student’s *t*-test was used to identify significant differences compared to the control (^*^p < 0.05, ^**^p < 0.01).

The effect of blocking ethylene signal transmission by 1-MCP on CBG expression and CAP contents was studied. The 1-MCP treatment remarkably reduced the expression of *PAL*, *C4H*, *COMT*, *pAMT*, and *CS* genes (Figure 7A). The 1-MCP treatment significantly reduced the content of CAPs (Figure 7B). The results indicated that ethylene signal transduction was involved in the regulation of CAP biosynthesis.

### Effects of PZA on CBGs Expression and CAPs Contents

The results indicated that after pepper fruits were treated with ethylene biosynthesis inhibitor PZA for 12 h, the expression of *ACC oxidase-2* (*ACO2*) gene was extremely reduced (Figure 7C); expression of CBGs such as *PAL*, *C4H*, *COMT*, *pAMT*, and *CS* was inhibited (Figure 7C); CAPs contents were also obviously reduced (Figure 7D); and the endogenous ethylene release rate was conspicuously reduced (Figure 7E). This indicated that the content of endogenous ethylene significantly affected biosynthesis of CAPs.

### The Effect of *CcERF2*-Silenced on CAPs Content and Metabolic Pathways

**Figure 8:**
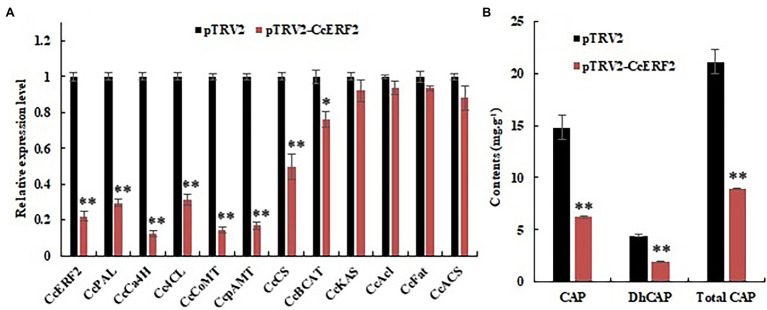
Expression level of CBGs and *CcERF2*, relative contents of CaP, DhCaP, and CAPs in the control (empty vector) and *CcERF2*-silenced fruit. **(A)** Expression level of CBGs and *CcERF2* in the control (empty vector) and *CcERF2*-silenced fruit. Fruits at 24 DAP was sampled to perform the expression level of CBGs and *CcERF2*. The relative expression level of the CBGs and *CcERF2* in the control were set to 1, and that of in the *CcERF2*-silenced fruit were measured relative to that in the control. **(B)** Relative contents of CaP, DhCaP, and CAPs in the control (empty vector) and *CcERF2*-silenced fruit at 34 DAP. The experiments were replicated three biological times and three technical times. Data are expressed as the mean ± SD (*n* = 9). Student’s *t*-test was used to identify significant differences compared to the control (^*^p < 0.05, ^**^p < 0.01).

The VIGS vector pTRV2–*CcERF2* was constructed using a vector derived from tobacco rattle virus (TRV), and a *CcERF2*-silenced experiment was performed. The 24-DAP fruits of SL08 were used for the study. It was demonstrated that *Agrobacterium* infection with an empty pTRV2 vector resulted in distinctive changes in expression level of CBGs in pepper fruits (Abraham-Juárez et al., 2008). Compared with empty vector plants, the fruits infected with the pTRV2–*CcERF2* construct showed a distinctive reduction in *CcERF2* expression (22.4% of the empty vector; Figure 8A). Consistent with the expression level of *CcERF2*, CBGs in silent plants were also significantly downregulated. In the *CcERF2*-silent placenta, the expression of genes derived from fatty acid metabolism pathways (such as *CcKAS*, *CcACL*, *CcFAT*, and *CcACS*) only slightly changed. However, expression of genes derived from the phenylpropane pathway (such as *CcPAL*, *CcCa4H*, *Cc4CL*, *CcComt*, and *CcpAmt*) and *CcCS* underwent significant changes, and the genes whose expression was altered were consistent with the transcription level of *CcERF2* (Figure 8A).

The CAPs contents of the pepper fruits infected with the empty pTRV2 vector were similar to those of uninfected plants (Abraham-Juárez et al., 2008). The 34-DAP fruits were used to study the effect of *CcERF2* silencing on the CaP and DhCaP contents. Compared with fruits infected with empty pTRV2, the CaP and DhCaP contents in fruits infected with pTRV2–*CcERF2* were significantly reduced by 74.2 and 73.0%, respectively (Figure 8B). The above results strongly supported that *CcERF2* regulated certain CBGs to control CAPs biosynthesis.

## Discussion

Plant secondary metabolism comprises the life activity, formed in the long-term evolutionary process. The secondary metabolites are not only associated with the regulation of plant growth, but also allow plants to effectively deal with all kinds of stresses, such as fungi, pests, and herbivores (Dangl and Jones, 2001). The distribution of secondary metabolites is usually specific to species, organs, and tissues; CAPs are uniquely synthesized in the fruit placenta of *Capsicum* and have obvious specificity (Mazourek et al., 2009). A variety of stress conditions (such as high temperature, hydropenia, and herbivore invasion) can induce the production of secondary metabolites, and this process is affected by a lot of plant endogenous hormones (such as ethylene). Studies have found that low temperature promotes the accumulation of CAPs in pepper fruits (Keyhaninejad et al., 2014; Arce-Rodríguez and Ochoa-Alejo, 2017); drought induces biosynthesis of CAPs (Phimchan et al., 2014); and injury significantly increases the CAPs content of pepper fruits (Arce-Rodríguez and Ochoa-Alejo, 2017). Plants under stress (such as high temperature, hydropenia, and herbivore attack) usually show an increase in ethylene content, which can increase the formation of secondary metabolites. Ethylene plays a vital factor in response to all kinds of stresses (Fang et al., 2014; Hu et al., 2020). However, the effect of CAPs biosynthesis induced by ethylene needs further study.

We investigated the mechanism of ethylene on the expression of *CcERF2* and CBGs. After fruits of line SL08 were treated with ethephon, expressions of *CcERF2* and CBGs were significantly increased, equivalent to 2.1–110.3 times that of the control. However, the response pattern of each gene induced by ethephon slightly differed (Figure 5). Many studies have indicated that ethephon can upregulate the expression of multiple genes in the phenylpropane metabolic pathway, thereby promoting biosynthesis of flavonoids, anthocyanins, rutin, lignin, and procyanidins (El-Kereamy et al., 2003; Buer et al., 2006; Hossain et al., 2009; Pan et al., 2019; Hu et al., 2020; Paul et al., 2020; Zhao et al., 2020). Our experimental results are similar to those reports.

Most AP2/ERF family proteins have conserved AP2 domains, and it was reported that this type of TF can regulate the synthesis of plant secondary metabolites (Paul et al., 2020). These AP2/ERF factors seem to play a similar role in regulation metabolic genes (Hu et al., 2020). In this paper, the AP2/ERF TF *CcERF2*, which can be used as a transcription activator to regulate CAPs biosynthesis was identified. There were 43 motifs obtained by analyzing the motif significance of the CcERF2 protein. On the whole, the predicted motifs differed within the same family, but the conserved motifs in the same subgroups were almost the same. That is, the closer the related species, the more similar were the motifs. The peppers all contained 13 conserved motifs (1–8, 11, 12, 17, 19, and 22), of which motifs 1, 3, and 5 covered the conserved AP2 structural domain. Our results showed that the function of CcERF2 was convergent and divergent among different plant species.

Various genes related to CAPs biosynthesis have been studied (Aluru et al., 2003). However, very few TFs associated with CAPs biosynthesis have been isolated and characterized, except for some MYB TFs (Arce-Rodríguez and Ochoa-Alejo, 2017; Sun et al., 2019, 2020; Zhu et al., 2019). We need to identify some TFs, especially AP2/ERF family TFs, which can be used as positive regulators to promote accumulation of CAPs. In the current study, based on transcription data, the AP2/ERF TF *CcERF2* was selected for further analysis, because *CcERF2* had a similar expression pattern to the CBGs in the transcriptome data. Silencing *CcERF2* downregulated the expression of CBGs, especially the expression level of genes associated with the phenylpropane pathway, and therefore reduced its CAPs content. The results showed that *CcERF2* was involved in regulating this metabolic process.

The 1-MCP is an ethylene receptor inhibitor (Sisler and Serek, 1997; Huber, 2008). It can effectively inhibit ethylene signal transduction (Du et al., 2021). A large number of studies have shown that 1-MCP can affect the maturation, senescence, and secondary metabolism of non-climacteric fruits by blocking ethylene signal transduction (Gómez-Lobato et al., 2012; Li et al., 2016; Zhang et al., 2018). Our research results showed that 1-MCP significantly reduced the expression of some capsaicin biosynthesis genes, and obviously reduced the content of CAPs in fruits.

The PZA is a new type of ethylene biosynthesis inhibitor that acts by inhibiting expression of *ACO2* (Sun et al., 2017). Our research showed that PZA obviously reduced *ACO2* expression in pepper fruits and remarkably reduced ethylene production. At the same time, PZA treatment greatly reduced the expression of some genes related to capsaicin biosynthesis, thereby reducing the content of CAPs.

## Conclusion

This study showed strong evidence that ethylene induced the expression of *CcERF2* and CBGs, and 1-MCP and PZA treatments caused a decrease in expression of CBGs and a sharp decrease in content of CAPs in fruit. It also demonstrated that *CcERF2* could promote CAP biosynthesis in *Capsicum*. It is necessary to further study whether *CcERF2* acts directly on the promoter of CBGs of the CAP pathway or through formation of a complex with other TFs.

## Data Availability Statement

The original contributions presented in the study are publicly available. This data can be found at: National Center for Biotechnology Information (NCBI) BioProject database under accession number PRJNA789050.

## Author Contributions

MD and KZ conceived and designed the study. JL, XZ, ZL, HZ, JH, and HW performed the research. ZW, HZ, XZ, and JW analyzed the data. MD, XZ, and JW prepared the paper. All authors contributed to the article and approved the submitted version.

## Funding

This study was supported by the Natural Science Foundation of China (Grant Nos. 31560556 and 31160394) and the Major Science and Technology Projects in Yunnan Province (2018BB020).

## Conflict of Interest

The authors declare that the research was conducted in the absence of any commercial or financial relationships that could be construed as a potential conflict of interest.

## Publisher’s Note

All claims expressed in this article are solely those of the authors and do not necessarily represent those of their affiliated organizations, or those of the publisher, the editors and the reviewers. Any product that may be evaluated in this article, or claim that may be made by its manufacturer, is not guaranteed or endorsed by the publisher.

## Footnotes

^1^ www.ncbi.nlm.nih.gov

## Supplementary Material

Supplementary Figure S1 Capsaicin (CaP), dihydrocapsaicin (DhCaP), and total capsaicinoids (CAPs) contents determination in inbred line SL08 and H19 fruits. Fifteen fruits were sampled for the measurement of the contents of CAP, DhCAP, and total CAPD at each developmental stage. The experiments were replicated three biological times and three technical times. Data are expressed as the mean ± SD (*n* = 9).
