# Supplementary material for: Ethylene-Inducible AP2/ERF Transcription Factor Involved in the Capsaicinoid Biosynthesis in Capsicum
Source: Front Plant Sci. 2022 Mar 3;13:832669. doi: 10.3389/fpls.2022.832669 (PMC8928445; doi:10.3389/fpls.2022.832669)
Supplement: Supplementary file 1 [file Table_1.DOCX]

| Primer name | Primer sequences |  |
| --- | --- | --- |
| CcERF2-F1 | TTCCCACTCACAACAAAACCAA | *CcERF2* clone |
| CcERF2-R1 | AGAAACCCATTCTCCTAAACCG |  |
| CcERF2-F2 | AGCCCAAACGAACTCAACCC | *CcERF2* expression |
| CcERF2-R2 | CAAAGCTCCATCAAGCCACC |  |
| CcPAL-F | TTTGCCTATGCTGATGATACCTG | *CcPAL* expression |
| CcPAL-R | GCTGTTCACATTCTTCTCGCTTT |  |
| CcCa4H-F | TCAGATTCCTTCCATTCGGT | *CcCa4H* expression |
| CcCa4H-R | CTTTCTCCGTGGTGTCGAG |  |
| Cc4CL-F | CTAGACTGGCTGCTGGTGTC | *Cc4CL* expression |
| Cc4CL-R | TCGCCCGTTGTCAAACTCAG |  |
| CcCoMT-F | AAACAAGCCATAGCCTAACTCAAAC | *CcCoMT* expression |
| CcCoMT-R | AAGTAGCAAGAAGCCTAAACATTCG |  |
| CcpAMT-F | TTTCATTGCCGAACCAGTC | *CcpAMT* expression |
| CcpAMT-R | GTCCCAAGTCTTCCAAATCCA |  |
| CcCS-F | CGCACAAGATTGGTGATGG | *CcCS* expression |
| CcCS-R | TTCTGTACGCACTCGTTGAGAT |  |
| CcBCAT-F | AAAGCGTTTAGAAGAGAGGATGG | *CcBCAT* expression |
| CcBCAT-R | GACAAGGAATGTGTACTCAGGTG |  |
| CcKAS-F | ATGAGTTTGGTAGATGCGGGA | *CcKAS* expression |
| CcKAS-R | CGGTGTCAATTGTAACCTGAGG |  |
| CcFAT-F | ACCTCGTAACACCTAACAATAAACTTT | *CcFAT* expression |
| CcFAT-R | AGAGAGAGTAAGAGTAAGCAGCAAGT |  |
| CcACL-F | ATCTCTTCCTTCAAGCACAACCA | *CcACL* expression |
| CcACL-R | TCCTCAAGTCCCATGACAATCTC |  |
| CcACS-F | CCAAACCAACACCTCCAAAC | *CcACS* expression |
| CcACS-R | CCAGCAAGCGGATAGAACA |  |
| CcERF2-F3 | GGAATTCCAGCCCAAACGAACTCAACCC | *CcERF2* VIGS |
| CcERF2-R3 | CGGGATCCCGCAAAGCTCCATCAAGCCACC |  |
| CcERF2-F4 | GCTCTAGAGCATGTGTGGTGGAGCAATTCT | *CcERF2* Subcellular localization |
| CcERF2-R4 | CGGGATCCCGAACTACATTATAACTAGGTTG |  |
| CcERF2-F5 | CGGGATCCATGTGTGGTGGAGCAATTCT | *CcERF2* transcriptional activation analysis |
| CcERF2-R5 | CGGAATTCAACTACATTATAACTAGGTTG |  |
| ACO2-F | GCAATGAAGGCTATGGAGACTA | *ACO2* transcriptional activation analysis |
| ACO2-R | TGACGCATCAAGAAACACAAG |  |
| β-ACTIN -F | TGCAGGAATCCACGAGACTAC | *β-ACTIN* expression |
| β-ACTIN -R | TACCACCACTGAGCACAATGTT |  |

Table S1 Primers used in this study
