# Supplementary figures and images for: Ethylene-Inducible AP2/ERF Transcription Factor Involved in the Capsaicinoid Biosynthesis in Capsicum
Source: Front Plant Sci. 2022 Mar 3;13:832669. doi: 10.3389/fpls.2022.832669 (PMC8928445; doi:10.3389/fpls.2022.832669)

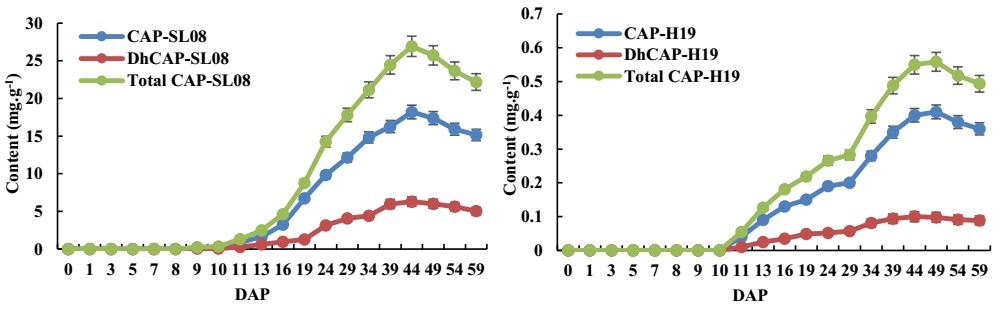

Supplement: Supplementary Figure S1 — Capsaicin (CaP), dihydrocapsaicin (DhCaP), and total capsaicinoids (CAPs) contents determination in inbred line SL08 and H19 fruits. Fifteen fruits were sampled for the measurement of the contents of CAP, DhCAP, and total CAPD at each developmental stage. The experiments were replicated three biological times and three technical times. Data are expressed as the mean ± SD (n = 9). [file Image_1.JPEG]
